# Shallow Graph Convolutional Network for Skeleton-Based Action Recognition

**DOI:** 10.3390/s21020452

**Published:** 2021-01-11

**Authors:** Wenjie Yang, Jianlin Zhang, Jingju Cai, Zhiyong Xu

**Affiliations:** 1 Key Laboratory of Optical Engineering, Institute of Optics and Electronics, Chinese Academy of Sciences, Chengdu 610209, China; yangwenjie17@mails.ucas.ac.cn (W.Y.); xueman1999@163.com (J.C.); xzy158@163.com (Z.X.); 2 School of Electronic, Electrical and Communication Engineering, University of Chinese Academy of Sciences, Beijing 100049, China

**Keywords:** activity recognition, graph convolution network, skeleton sequence

## Abstract

Graph convolutional networks (GCNs) have brought considerable improvement to the skeleton-based action recognition task. Existing GCN-based methods usually use the fixed spatial graph size among all the layers. It severely affects the model’s abilities to exploit the global and semantic discriminative information due to the limits of receptive fields. Furthermore, the fixed graph size would cause many redundancies in the representation of actions, which is inefficient for the model. The redundancies could also hinder the model from focusing on beneficial features. To address those issues, we proposed a plug-and-play channel adaptive merging module (CAMM) specific for the human skeleton graph, which can merge the vertices from the same part of the skeleton graph adaptively and efficiently. The merge weights are different across the channels, so every channel has its flexibility to integrate the joints. Then, we build a novel shallow graph convolutional network (SGCN) based on the module, which achieves state-of-the-art performance with less computational cost. Experimental results on NTU-RGB+D and Kinetics-Skeleton illustrates the superiority of our methods.

## 1. Introduction

Action recognition is a crucial task in computer vision, which has broad applications such as man–machine interaction, video surveillance, and intelligent health caring [1,2]. It has attracted interest from many researchers. However, one matter of video-based action recognition method is massive computing consumption. Biological studies have shown that even without detailed appearance information such as frames in videos, a few joints’ locations can effectively represent human action. As a result, many researchers focus on skeleton sequence data, and lots of achievement emerges.

Earlier methods generate a representation of human action simply by the hand-crafted features [3,4,5,6,7]. Recently, deep-learning-based methods have achieved considerable performance in many computer vision tasks. Many researchers have tried to use convolutional neural networks (CNNs) [8,9,10,11] and Recurrent Neural networks (RNNs) [12,13,14,15] to recognize human action from skeleton data. Most of them represent the skeleton data as a type of grid-like structure such as a vector sequence or a 2D grid, but it cannot well express the natural structure information of the human skeleton.

ST-GCN [16] introduced graph convolutional network (GCN) to skeleton-based action and use the spatial-temporal graphs to express the structure information of the human skeleton. After that, many methods [17,18,19] follow the research and use GCN for skeletal action recognition. Shi et al. [20] proposed the adaptive graph convolutional network with an adaptive adjacent matrix that enables the network to learn the topology of the human skeleton automatically, it extracts second-order information (lengths and directions of bones) of skeleton data as an extra input stream of the network to strengthen its discrimination.

However, there are still some problems with the existing GCN-based methods. In most of those GCN-based methods, spatial graph size is fixed among all the layers. We argue that the fixed graph size limits the ability to exploit global and semantic information and cause unnecessary redundancies. One essential factor involved in the model’s ability to exploit the global information is the receptive field, which is defined as the region in the input space that the model’s feature is looking at. Existing GCN-based methods mostly choose to stack more layers and make the network deeper, which only increases the receptive field size linearly by theory [21]. Stacking too many layers could increase both the model’s complexity and the risk of suffering from the over-fitting problem. Furthermore, the fixed graph size would also cause many redundancies in the features of deep layers. The redundancies can hinder the model from extracting exactly important discriminative features.

To address those issues, we proposed an efficient channel merging module (CAMM) that is specific for the skeleton graph data. The module is proposed to merge the vertices from the same part of human skeletons. We assign different weights across the channels, so every channel has its flexibility to integrate the joints. This setting is intended to make every channel focus on different vertices in the same part of the graph and get more plentiful features. We then build a shallow graph convolutional network (SGCN) based on the CAMM, which only has six layers but reaches a better performance. A multi-scale feature fusion strategy is introduced to integrates the local detailed information and global semantic information. Since the temporal receptive field is also reduced by reducing layers, we use a temporal dilated convolution as compensation for it. Our model has fewer parameters and little computational cost yet a higher performance than the existing deeper models. We evaluate the proposed method on two benchmarks, NTU-RGB+D [13] and Kinetics-Skeleton [16,22]. The proposed model achieves a better performance with the state-of-the-art on both datasets. The contributions of this paper are manifold:

- We proposed a new plug-and-play channel adaptive merging module (CAMM) to merge the vertices from the same part of the skeleton graph adaptively and efficiently. It can flexibly broaden the receptive field with different graph network depth and reduce the features’ redundancies.
- We build a shallow graph convolutional network (SGCN) based on the CAMM. We deploy the multi-scale feature fusion strategy and temporal dilated convolution to improve the model’s robustness, which enables the network to exploit global semantic information and preserve the local detailed features simultaneously.
- Experimental results on two large-scale large-action recognition datasets: NTU-RGB+D and kinetics-skeleton, demonstrate that our method achieves state-of-the-art performance on both of evaluated datasets.

## 2. Related Works

Human action recognition based on human skeleton sequences is extensively explored during recent years. Researchers usually represent the skeleton data with 2D or 3D human joint coordinates of all frames. Traditional human skeletal action recognition methods generally design hand-crafted features. Xia et al. [3] used the 3D joint point histogram and discrete Markov model to represent human action. Keceli et al. [4] extract angle and displacement information of skeleton joint points as the human action features. Gowayyed et al. [5] extended the histogram of oriented displacements (HOD) to 3 dimensions as a representation of actions. Vemulapalli et al. [6] use the relative geometry between every pair of body parts as a description of human action. Fernando et al. [7] use the parameters of ranker as the representation of actions. Due to the abundant data and increased computing power, deep learning has become increasingly popular in the past few years. Deep-learning-based methods have achieved considerable performance in many computer vision tasks. Many researchers recognize human action from skeleton data by [8,9,10,11] and Recurrent Neural networks (RNNs) [12,13,14,15]. For CNN-based methods, Kim et al. [8] propose a temporal convolutional neural network to learn spatial-temporal representations for human actions. Ke et al. [9] transform each skeleton sequence into three clips generated from 3 channels of the cylindrical coordinates and use a multi-task learning network to recognition actions. Liu et al. [10] use the transform to eliminate the effect of view variations locations visualize skeleton sequences as a series of color images to encode the spatial-temporal information. For RNN-based methods, Du et al. [12] divide the human skeleton into five parts and separately feed them to five subnets. The outputs from different subnets are hierarchically fused to generate a representation of the actions. Liu et al. [14] propose a spatio-temporal long short-term memory network. It extends the traditional LSTM-based learning to two domains, the temporal domain, and the spatial domain. Li et al. [15] propose independently recurrent neural network to recognize actions in human skeletons. Though some improvement has been made, there is still one problem. Most of them represent the skeleton data as a grid-like structure such as a vector sequence or a 2D grid. It is not optimal because both the vector sequence and the 2D grid cannot effectively use the human skeleton’s natural structure information.

In recent years, graph convolution operation was proposed to process some tasks based on graph-structure data. As human joints have a natural structure and connections, the skeleton data can be represented by a graph in which every vertex represents a human joint. The first application of graph convolution in skeletal action recognition is the ST-GCN [16], which represents the full skeleton sequence as a spatial-temporal graph, the same joints of consecutive frames are connected. The coordinate vector of each joint is set as the attribute of the corresponding vertex. Therefore, in NTU-RGB+D [13], the input skeleton sequence has three channels, while two channels in the Kinetics-Skeleton [16,22]. After that, many methods [17,18,19,20] follow the research and use GCN for skeletal action recognition. Shi et al. [20] proposed to add an adaptive adjacent matrix to exploit the co-relation between joints that are not directly connected by in the predefined human-body-based graphs. It extracts second-order information (lengths and directions of bones) as an extra input stream of the network to strengthen its discrimination. Song et al. [18] propose a model that can reuse the information implied in unactivated joints. Li et al. [17] introduce an encoder-decoder structure to capture action-specific latent dependencies. Zhu et al. [19] use a fully adaptive adjacent matrix that enables the network to learn the topology of the graph automatically. In the above GCN-based methods, spatial graph size is fixed among all the layers. We argue that the fixed graph size would hinder the model from broadening the receptive fields, exploiting the global information, and cause unnecessary redundancies. Thus, a suitable way is needed to adjust the graph size, broaden the spatial receptive fields, and remove redundant features.

## 3. Method

This section will introduce the details of our channel adaptive merging modules, the multi-scale fusion strategy architecture, and other components of the proposed shallow graph convolution network (SGCN).

### 3.1. Overview

**Figure 1:**
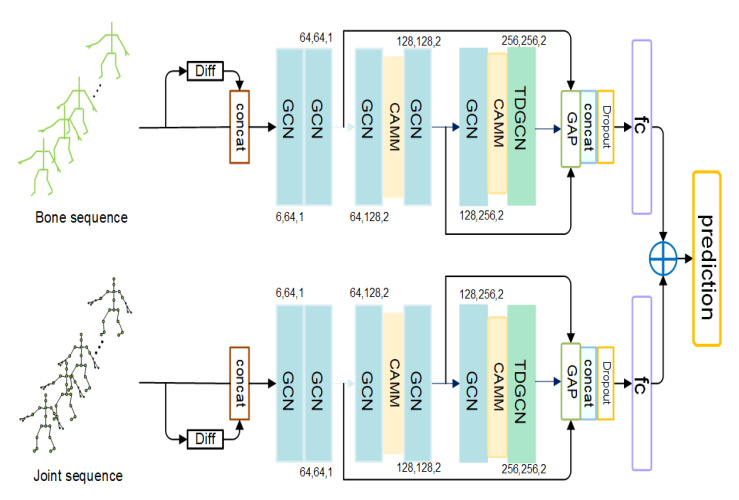
The illustration of our proposed SGCN architecture. GCN is the spatial-temporal graph convolution block. Its three numbers represent the number of input channels, output channels, and the temporal stride. CAMM is the channel adaptive merging module. TDGCN is the spatial-temporal graph convolution block with temporal dilated convolution. GAP means global average pooling. Features from different layers are combined to get a steady representation of the action.

An overview of our proposed SGCN is illustrated in Figure 1. The proposed SGCN has two streams for bone and joint data of the human skeleton, respectively. Each stream has the same architecture but trained independently. We combine the scores of two streams to get a final prediction during the test period in a weight-sum manner while testing.

The joint data is the original coordinates sequences of the human joints. We follow the bone modeling approach in [20]. Each bone is represented as a vector, given a bone with its source joint $v_{1}=\left(x_{1},y_{1},z_{1}\right)$ and its target joint $v_{2}=\left(x_{2},y_{2},z_{2}\right)$, the vector of the bone is calculated as $e_{v_{1},v_{2}}=\left(x_{2}-x_{1},y_{2}-y_{1},z_{2}-z_{1}\right)$. We can assign each bone with a unique target joint because there are no cycles on the skeleton graph. However, the number of joints is one more than the number of bones because there is no bone for the central joint to assign. Therefore, an empty bone is added with its value as 0 to the central joint. In this way, we can bind each bone with a unique joint to design the same graph and network for the bones and joints. The differences between every two adjacent frames are calculated as the motion sequence. We pad the first frame of motion sequence with 0 to ensure it has the same number of frames as the original joint or bone sequence. We concatenate it with the original data sequence as the input of one stream.

We employ a six-layer backbone based on graph convolution blocks to learn the representation of actions for every stream. The backbone is very light-weight, which only has almost one half layers than other existing GCN-based methods [16,17,18,19,20]. We embed two-channel adaptive merging modules (CAMM) behind the second and fifth graph convolutional block. Every CAMM can almost reduce the feature map’s spatial size by half, which significantly reduces redundant features and broadens the receptive field. We will introduce the details of the CAMM in Section 3.2. However, the simple adoption of CAMM may also damage useful local information from the previous layers. To move beyond this limitation, we design a multi-scale framework. The outputs of previous layers are transported to the final global average pooling layer to preserve the different level semantic information. After global average pooling, the features from different layers are concatenated and sent to the fully connected layer. We combine the scores of two streams in a weight-sum manner to get the final prediction. It is worth noting that the temporal receptive field is narrowed with the reduction of layers too. Therefore, we introduce the temporal dilated convolution in the last graph convolutional block to expand the temporal receptive field.

### 3.2. Channel Adaptive Merging Module

The fixed spatial graph size of graph convolution has two obvious disadvantages. On the one hand, the fixed spatial graph size will lead to unnecessary redundancies of features. It slows down the prediction of the model. Furthermore, the redundancies make it more difficult to get useful discriminative information. On the other hand, the model is hard to get enough receptive fields with a fixed spatial graph size, which prevents the network from getting high-level semantic information. Existing methods usually stack more layers for broader receptive fields. However, it can only slowly increase the receptive fields but give rise to the over-fitting problem. The non-local block deployed in [20] may help to broaden the receptive fields, but it has a high computational cost and cannot capture the correlations between part and part of the human skeleton.

**Figure 2:**
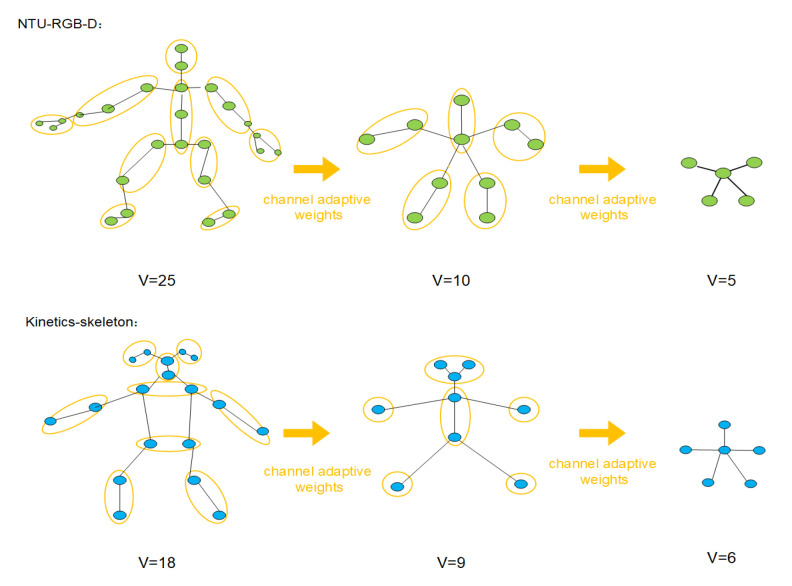
Illustration of the channel adaptive merging operation for the human skeleton graph of NTU-RGB+D and kinetic-skeleton. Vertices in one yellow circle are merged into one vertex in a weight-sum manner. Every channel has its weights to merge the vertices.

To solve those problems, we propose a channel adaptive merging module (CAMM) to realize the efficient merging operation of the human skeleton graph. We can naturally recognize that the human skeleton is a hierarchical structure. More precisely, the whole skeleton consists of several parts, and each can also be divided into smaller sub-parts. Therefore, we intuitively merge vertices in the same sub-part of the skeleton graph in a weight-sum manner. As shown in Figure 2, vertices in the same part of the skeleton are merged into one vertex. The CAMM will merge the connection from and to the vertices in the same part and remove the connection between those vertices in the same part.

#### 3.2.1. The Merging Operation

The skeleton data for a single frame can be viewed as a graph, represented as G=(V,E), it includes the vertex set $V=\{v_{i}|i=1,2,...,N\}$ and the edge set $E=\{e_{ij}|i=1,2,...,N,j=1,2,...,N\}$. *N* is the number of joints in the human skeleton, the merging operation in one channel can be formulated as:

(1) $$v_{out}\left(k\right)=\frac{1}{N_{k}}\sum_{i\in part\;k}v_{in}\left(i\right)*w_{k}\left(i\right)$$

where $N_{k}$ is the number of vertices in the *k*th part. $v_{in}\left(i\right)$ is the *i*th vertex of the input graph. $v_{out}\left(k\right)$ is the *k*th vertex of the output graph. $w_{k}\left(i\right)$ is the trainable weights for contribution of joint *i* in part *k*. If the different merge weights between channels are also taken into account, Equation (1) can be changed to:

(2) $$v_{out}^{c}\left(k\right)=\frac{1}{N_{k}}\sum_{i\in part\;k}v_{in}^{c}\left(i\right)*w_{k}^{c}\left(i\right)$$

where *c* is the index of the channel in the feature map. Please note that the CAMM does not change the number of channels in the feature map. Thus, its inputs channels and outputs channels share a one-to-one mapping. If we construct the weight matrix $W^{c}\in R^{N\times N^{\prime }}$ for channel *c* like:

(3) $$W^{c}\left(i,k\right)=\left\{\begin{array}{ccc} \frac{1}{N_{k}}*w_{k}^{c}\left(i\right) & , & i\in part\;k,\\ 0\; & , & \mathrm{otherwise}. \end{array}\right.$$

where *N* and $N^{\prime }$ are the number of vertices before and after the merging. Please note that $N^{\prime }$ is much smaller than *N*. Then, we can express Equation (2) in matrix form as:

(4) $$V_{out}^{c}=V_{in}^{c}*W^{c}$$

where $V_{in}^{c}$ is the vertex set of the input graph in channel *c*, $V_{out}^{c}$ is the vertex set of the output graph in channel *c*, $W^{c}$ is the weight matrix of channel *c*. In this way, every channel has its own weight to merge the vertices in the same part of the graph. Comparative experiments show that channel-wise weight has better performance than channel-shared weights, which will be introduced in Section 4.

#### 3.2.2. Partition Details of Skeleton

**Figure 3:**
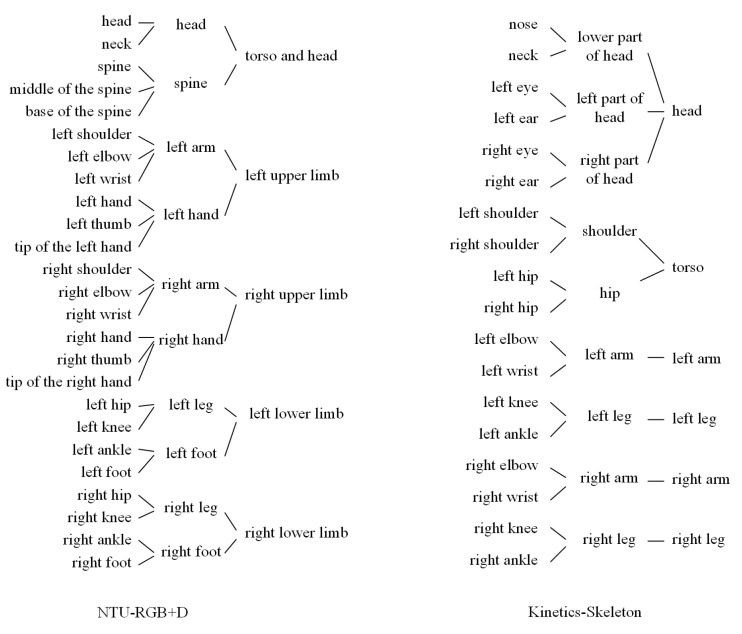
The illustration of the partition strategies for the human skeleton graph of NTU-RGB+D and Kinetic-Skeleton.

**Figure 4:**
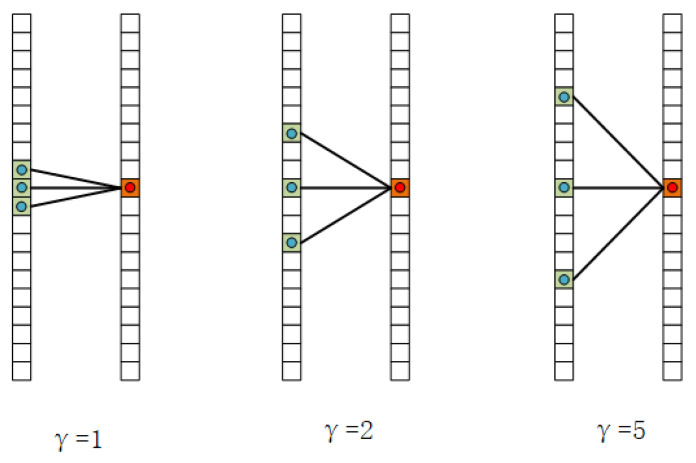
The illustration of temporal dilated convolution, the kernel is 3, γ means the dilation factor.

Here we introduce the details of the partition strategies applied on the NTU-RGB+D [13] and Kinetics-Skeleton [16,22]. As shown in Figure 3, we design the partition strategies according to the natural human skeleton structure and the dataset joints assignments. On the one hand, we intend to make every part or sub-part has its semantics properties. We argue that semantically splitting the skeleton can help the model better preserve the skeletons’ structure information. Therefore, almost all parts or sub-parts in Figure 4 have the semantics properties. On the other hand, we are trying to avoid merging too many vertices of the same part at once. Rapid reduction of vertices may lead to disruption of local information. For example, in the Kinetics-Skeleton dataset, we merge the vertices near the head by two steps instead of one because there are six vertices near the head, which is more than other skeleton parts.

For the bone stream, the vertices’ attributes are the second-order information, namely bones’ lengths and directions. As mentioned in Section 3.1, we bind each bone with a unique joint and design the same graph and network for the bones and joints. Therefore, we also use the same skeleton partition strategy and merging operation for both the joint and bone streams.

### 3.3. Multi-Scale Feature Fusion

As we all know, the actions such as “hopping”, “stand up”, and “punch” may need the whole body to complete. So, the model must get global information of the entire skeleton to have good discrimination of those actions. But actions such as “writing”, “nod head”, “type on a keyboard” only need a small part of the body. Thus, the model needs local detailed information from certain parts of the skeleton to recognize those actions. Existing GCN-based methods usually use the feature map from the last layer to generate action class scores. The local information in the shallow layers is hard to reach the last layer. The adoption of CAMM can also block local features from reaching deeper layers.

We design a multi-scale framework to solve the problem, as shown in Figure 1. We transfer the features from the second and fifth layer to the final global average pooling layer with two lateral connections. The temporal and spatial size of features is reduced to 1×1 by the global average pooling. Then we concatenate the features from different layers in the channel dimension to form a long feature vector. It contains both the global and local information, which is a steadier representation of the actions. This structure can also help the network alleviate the vanishing gradient problem and exploding gradient problem. The supervision can directly reach the shallow through the lateral connection. The experimental results show that the performance of the method promotes clearly owing to the multi-scale fusion. We will discuss the details of the experiments in Section 4.

### 3.4. Temporal Dilated Convolution

As we introduced above, we use a shallower network with inherently smaller receiver fields than the deeper network. Therefore, we need to expand the receptive field of our model. The spatial receptive field is expanded with CAMM’s application, so we only need to focus on expanding the temporal receptive fields. To this end, we introduce the temporal dilated convolution to the last GCN layers. Dilated convolution was first proposed by Holschneider et al. [23] for the analysis of a wavelet decomposition algorithm. It is referred to as “convolution with a dilated filter”. Yu et al. [24] applied the dilated convolutions to semantic segmentation tasks. It supports exponentially expanding receptive fields without losing resolution or coverage. Most researchers use the 2D dilated convolution, and it is never used in the skeleton-based action recognition task. We introduce it into the skeleton-based action recognition task. Moreover, we change it to a 1D form because only the temporal dimension of the skeleton sequences is a grid-like structure. Let {f(t)|t=1,…,T} be the input sequence, {k(s)|s=1,…,S} be the parameters of convolution kernel. The output of 1D discrete convolution can be formulated as:

(5) $$g\left(t_{0}\right)=\sum_{s\in S}f\left(t_{0}+s\right)k\left(s\right)$$

For every output point $t_{0}$ the sampling location is $\left\{t_{0}+s|s=1,\ldots ,S\right\}$. If we use dilated convolution, the Equation (5) can be converted to:

(6) $$g\left(t_{0}\right)=\sum_{s\in S)}f\left(t_{0}+\gamma s\right)$$

where γ is the dilation factor. For every output point $t_{0}$, the sampling location is $\left\{t_{0}+\gamma s|s=1,\ldots ,S\right\}$. The number of sampling points remains the same, but the sampling range is expanded. We can find from Equation (6) that the relationship of sampling range *R*, the kernel size *S*, and the dilation factor γ of dilated convolution, which as formulated as:

(7) R=γ×(S−1)+1

We can regard the sampling range of temporal convolution as the temporal receptive fields. We apply the dilated convolution at the last GCN and form a temporal dilated graph convolution block to broaden the temporal receptive fields.

## 4. Experiments

We conduct our experiments on the same two large-scale action recognition datasets to verify the performance and efficiency of SGCN. Namely NTU-RGB+D [13] and Kinetics-Skeleton [16,22]. To investigate the proposed model’s effectiveness, we perform exhaustive ablation studies on NTU-RGB+D due to its smaller size than Kinetics-Skeleton. Then, we train and evaluate the proposed SGCN both on NTU-RGB+D and Kinetics-Skeleton to verify the generality. Furthermore, we compare the performance of SGCN with other state-of-the-art approaches.

### 4.1. Datasets

#### 4.1.1. NTU-RGB+D

NTU-RGB+D [13] is a famous and widely used dataset, consists of 56,880 action clips and 4,000,000 frames of 60 action classes, including daily, mutual, and health-related actions. They invited 40 volunteers for the data collection. Three cameras are used at the same time to capture three different horizontal views of the same action. For each setup, the three cameras located at the same height but from three different horizontal angles: $-45^{\circ },0^{\circ },+45^{\circ }$. The dataset provides the 3D locations of 25 joints of each subject. Each video contains up to 2 persons. The 3D joint locations were captured by Kinetic depth sensors. The benchmark evaluations include two evaluation protocols.

1. Cross-Subject (CS): The subjects are divided into two groups: training group and testing group, every group has 20 subjects. The training set has 40,320 videos from subjects in the training group, and the testing set has 16,560 videos of subjects in testing groups.
2. Cross-View (CV): The training set has 37,920 videos from cameras 2 and 3, and the testing set has 18,960 samples from camera 1.

We follow the cross-subject and cross-view evaluation protocols and report the top-1 accuracy under both protocols.

#### 4.1.2. Kinetics

Kinetics [22] is a Large-scale, high-quality human action recognition dataset, consists of 300,000 YouTube video clips of 400 classes. The video clips split into a training set (240,000 clips) and a validation set (20,000 clips). It is a video dataset, so there are no skeleton data provided. Yan et al. [16] estimate the coordinates and confidence of 18 joints of every subject in each frame with the OpenPose toolbox [25]. They [16] select two people with the highest average joint confidence for multi-person clips. We use the data (Kinetics-Skeleton) released by them as our benchmark to test the performance of our model. We report the top1 and top5 accuracies on the validation set.

### 4.2. Experimental Setting

All our experiments are conducted on the Pytorch deep learning framework [26]. We use cross-entropy as the loss function, choose stochastic gradient descent (SGD) with Nesterov momentum (0.9) as the optimization strategy, set weight-decay to 0.0001, the batch size is 64. We also choose the two-stream framework of joints and bone sequence. Adjacent frames’ difference is calculated and concatenate with the original joint or bone sequence as motion information. The graph-structure in the forepart of the network is the same as ST-GCN [20]. But it changed after channel adaptive merging, and we make some change that we showed in Figure 2. The first and second GCN’s temporal kernel size is set to 1 and 3, respectively. This setting keeps more local information, but the temporal kernel size of other layers is set to 9. For the NTU-RGB+D dataset, we apply cutting and zero-padding to make the number of people in each sample to be 2, the temporal length of every sample to be 300 frames. The dilation factor of TDGCN is set to 5; the fully connecting layer’s dropout rate is set to 0.4. The initial learning rate is 0.1 and decreases 10 times at the 30th and 40th epoch, and we train our model for 60 epochs. For the Kinetics-Skeleton dataset, we make each video contains 150 frame and two people by cutting and zero-padding. Those frames are randomly chosen from the input sequence and argument with random rotation and translation. The dilation factor of TDGCN is set to 5, and the dropout rate of the fully connecting layer is set to 0 because Kinetics-Skeleton is hard to fitting. The initial learning rate is 0.1 and decreases 10 times at the 45th and 55th epoch. We train our model for 65 epochs.

### 4.3. Ablation Study

We examine the effectiveness of our components in this section by experiments. In this section, our experiments only on the NTU-RGB+D dataset under the Cross-View (CV) protocol. Please note that we only train the model of joints sequence stream as default to ease the comparison except the experiment to examine the superiority of multi-modality framework.

#### 4.3.1. Channel Adaptive Merging

At first, we evaluate the necessity of the channel adaptive merging module and its channel adaptive weights. As introduced in Section 3.2, we apply the channel adaptive merging after the 2nd and 5th GCN layers. We manually delete the modules and show their performance in Table 1. The table shows that the channel-wise adaptive merging is beneficial to accuracy. Furthermore, we also set the weight matrix of the modules to be shared among all channels to construct a channel-shared merging module (CSMM). We embed it in the same location as CAMM, and its performance is also shown in Table 1. From the table, we can see that the model with CSMM and the model with CAMM has better accuracy than the model without merging operation, which proves the necessity of the merging operation. It can broaden the receptive fields and reduce the redundant features. Furthermore, the model with CAMM achieves the best accuracy, which implies the importance of the channel adaptive weights. By assigning different merging weights across the channels, we make every channel has its flexibility so that the model can get more plentiful feature.

**Figure 5:**
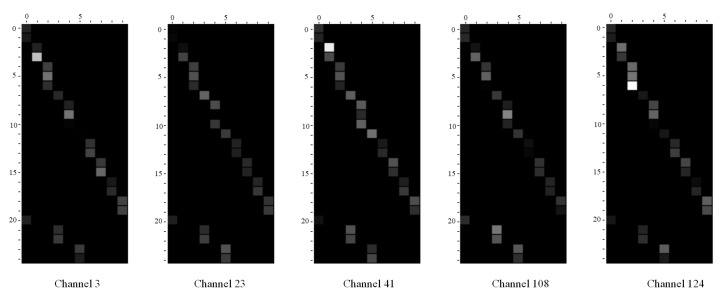
The Visualization of merging weights in CAMM. The horizontal coordinate is the index of output vertices, and the vertical coordinate is the index of input vertices. The channel is chosen randomly.

We visualized the fusion weights in some channels of the CAMM after the second layer. Then, we randomly choose five channels to show in Figure 5. We can see from the pictures that every channel has its attention for the joints in the same part. For example, for the third part (left arm), channel 124 pays more attention to joint 7 (left wrist), while other channels give a bigger weight to joint 6 (left elbow). Moreover, we can see that every channel also has its attention for the different parts. Channel 3 and channel 23 have a bigger weight for the second part (head and neck), channel 124 pays more attention to the third part, and the rest two channels have almost the same weight for every part. Thus, the model can focus on different parts simultaneously and capture the features for various actions. We can infer that the channel adaptive weights can capture the diverse features and help the model get a more reliable and stable action representation.

#### 4.3.2. Multi-Scale Features

As introduced in Section 3.3, we combine the output features from the 2nd, 4th GCN layers, and the final TDGCN to form a multi-scale feature vector that contains the different semantic level information. To prove the superiority of the approach, we manually remove the one or two lateral connections of the SGCN and then compare their performance with the model with the full multi-scale framework in Table 2. We can see from the table clearly that the model with the complete multi-scale framework outperforms others. Models with only one lateral connection will also perform better than a model with no lateral connections. We can infer that the combination of different semantic level features is helpful to action recognition in skeleton data. The multi-scale framework can preserve local detailed information and global semantic information at the same time.

#### 4.3.3. Different Dilation Factor

In the TDGCN, we applied temporal dilated convolutions, and we set the dilation factors to be 5 in this work. Here we focus on validating the effectiveness of temporal dilated convolutions and finding the most suitable dilation factors. We manually set the dilation factors to be 1, 3, 5, 7, respectively. Table 3 shows the top-1 accuracy of models with different dilation factors. When the dilation factor is 1, the dilated convolution can be regarded as the norm discrete convolution. We see on the table that the model’s performance is improved with the dilation factor’s growth when it is lower than 5. It implies that the temporal receptive field of expanded by the temporal dilated convolution. However, when the dilation factor increases to be greater than 5, the model’s performance decrease. These experiments indicate that suitable dilation factors could effectively broaden the receptive field and promote recognition accuracy. The dilation factor can be considered the temporal sampling rate on video, which would intuitively be closely related to the speed of actions and camera imaging speed. Via the dilation factor, we can flexibly adjust the networks’ temporal sampling rate to the actions’ actual speed.

#### 4.3.4. Multi-Modality Framework

We compare the performance of Js-SGCN (using joint input), Bs-SGCN (using bone input), and 2s-SGCN (using both) in Table 4 to prove the importance of the multi-modality framework. We can know from the table that the two-stream method outperforms either one-stream method.

## 5. Comparisons to the State-of-the-Art

To verify the generality of the proposed method, we compare SGCN on skeleton-based action recognition task with the state-of-the-art methods on the datasets of NTU-RGB+D and Kinetics-Skeleton. We use the methods based on handcraft-features [6,7], Recurrent neural network [12,13,15], convolutional neural network [8,9,11], and graph convolutional networ [16,17,18,19,20,27] for the comparison. The result of NTU-RGB+D is shown in Table 5 we report the top-1 accuracy for both cross-subject (CS) and cross-view (CV) protocols. And Table 6 shows the top-1 and top-5 accuracy of different state-of-the-art methods in Kinetics-Skeleton. We see that 2s-SGCN outperforms the other methods on both two datasets, which demonstrates our model’s effectiveness.

## 6. Conclusions

In this work, we propose a novel plug-and-play channel adaptive merging module (CAMM) specific for the human skeleton graph, which can effectively merge the joints in the same part of the graph. It can help the model to broaden the spatial receptive fields of the model, reduce the redundancies of features, and get more abundant features.

Then, we build a shallow graph convolutional network (SGCN) based on the CAMM. It has fewer layers but better performance than the existing GCN-based method for skeletal action recognition. To preserve global semantic features and the local detailed features simultaneously, we introduce the multi-scale feature fusion framework, which can adequately use the features with different semantic levels. Since the temporal receptive fields is reduced with the reductions of layers, we deploy a temporal dilated convolution to recover the temporal receptive fields. Experimental results on two large-scale action recognition datasets, NTU-RGB+D, and Kinetics-Skeleton prove our method’s effectiveness and superiority. Though our model has fewer layers, it achieves state-of-art performance on both datasets.

## Figures and Tables

**Table 1:** Comparisons of validation accuracy when the model with or without CAMM. ‘w/’ means ‘with’, ‘wo/’ means ‘without’.

Method	Accuracy (%)
SGCN wo/CAMM	94.7
SGCN w/CSMM	94.8
SGCN w/CAMM	95.2

**Table 2:** Comparisons of validation accuracy of the model with the different lateral connections. The number *i* in the second column means the model has a lateral connection from *i*th layer to the fully connected layer.

Method	Lateral Connection	Accuracy (%)
SGCN	/	94.9
SGCN	2	95.0
SGCN	4	94.8
SGCN	2, 4	95.2

**Table 3:** Comparisons of validation accuracy of the model with different dilation factors.

Method	Dilation Factors	Accuracy (%)
SGCN	1	94.7
	3	95.0
	5	95.2
	7	95.0

**Table 4:** Comparisons of validation accuracy with different input modalities.

Method	Accuracy (%)
Js-SGCN	95.2
Bs-SGCN	95.2
2S-SGCN	96.2

**Table 5:** Comparisons of the validation accuracy with state-of-the-art method on the NTU-RGB+D dataset.

Method	CS (%)	CV (%)
Lie Group [6]	50.1	52.8
H-RNN [12]	59.1	64.0
Deep LSTM [13]	60.7	67.3
PA-LSTM [13]	62.9	70.3
ST-LSTM [14]	69.2	77.7
STA-LSTM [28]	73.4	81.2
Ind-RNN [15]	81.8	88.0
TCN [8]	74.3	83.1
Clips + CNN + MTLN [9]	79.6	84.8
Synthesized CNN [10]	80.0	87.2
3scale-ResNet152 [11]	85.0	92.3
ST-GCN [16]	81.5	88.3
RA-GCN [18]	85.9	93.5
AS-GCN [17]	86.8	94.2
2s-AGCN [20]	88.5	95.1
Two-stream TL-GCN [19]	89.2	95.4
2s-SGCN (ours)	90.1	96.2

**Table 6:** Comparisons of the validation accuracy with state-of-the-art method on the Kinetics-Skeleton dataset.

Method	Top 1 (%)	Top 5 (%)
Method Top-1(Feature Enc. [7]	14.9	25.8
Deep LSTM [13]	16.4	35.3
TCN [8]	20.3	40.0
ST-GCN [16]	30.7	52.8
AS-GCN [17]	34.8	56.5
2s-AGCN [20]	36.1	58.7
Two-stream TL-GCN [19]	36.2	59.0
2s-SGCN (ours)	37.1	60.0

## Data Availability

Code of SGCN: https://github.com/kraus-yang/SGCN. Datasets: NTU-RGB+D: https://github.com/shahroudy/NTURGB-D. Skeleton-Kinetics: https://github.com/yysijie/st-gcn.
